# A GATA4/WT1 cooperation regulates transcription of genes required for mammalian sex determination and differentiation

**DOI:** 10.1186/1471-2199-9-44

**Published:** 2008-04-29

**Authors:** Yoko Miyamoto, Hiroaki Taniguchi, Frédéric Hamel, David W Silversides, Robert S Viger

**Affiliations:** 1 Ontogeny-Reproduction Research Unit, Centre de Recherche du Centre Hospitalier Universitaire de Quebec (CRCHUQ), Quebec City, QC, G1V 4G2, Canada; 2 Centre de Recherche en Biologie de la Reproduction (CRBR) and Department of Obstetrics and Gynecology, Laval University, Quebec City, QC, G1K 7P4, Canada; 3 Faculty of Veterinary Medicine, University of Montreal, St-Hyacinthe, J2S 7C6, Canada

## Abstract

**Background:**

In mammals, sex determination is genetically controlled. The *SRY *gene, located on Y chromosome, functions as the dominant genetic switch for testis development. The *SRY *gene is specifically expressed in a subpopulation of somatic cells (pre-Sertoli cells) of the developing urogenital ridge for a brief period during gonadal differentiation. Despite this tight spatiotemporal expression pattern, the molecular mechanisms that regulate *SRY *transcription remain poorly understood. *Sry *expression has been shown to be markedly reduced in transgenic mice harboring a mutant GATA4 protein (a member of the GATA family of transcription factors) disrupted in its ability to interact with its transcriptional partner FOG2, suggesting that GATA4 is involved in *SRY *gene transcription.

**Results:**

Although our results show that GATA4 directly targets the pig *SRY *promoter, we did not observe similar action on the mouse and human *SRY *promoters. In the mouse, Wilms' tumor 1 (WT1) is an important regulator of both *Sry *and Müllerian inhibiting substance (*Amh/Mis*) expression and in humans, *WT1 *mutations are associated with abnormalities of sex differentiation. GATA4 transcriptionally cooperated with WT1 on the mouse, pig, and human *SRY *promoters. Maximal GATA4/WT1 synergism was dependent on WT1 but not GATA4 binding to their consensus regulatory elements in the *SRY *promoter and required both the zinc finger and C-terminal regions of the GATA4 protein. Although both isoforms of WT1 synergized with GATA4, synergism was stronger with the +KTS rather than the -KTS isoform. WT1/GATA4 synergism was also observed on the *AMH *promoter. In contrast to *SRY*, WT1/GATA4 action on the mouse *Amh *promoter was specific for the -KTS isoform and required both WT1 and GATA4 binding.

**Conclusion:**

Our data therefore provide new insights into the molecular mechanisms that contribute to the tissue-specific expression of the *SRY *and *AMH *genes in both normal development and certain syndromes of abnormal sex differentiation.

## Background

In eutherian mammals, the gene responsible for triggering testis development, and hence male sex determination, is *SRY *(Sex Determining Region, Y chromosome) which encodes a putative transcription factor containing a high mobility group box DNA binding domain. SRY initiates the male pathway by triggering the differentiation of Sertoli cells in the genital ridge, the precursor of the developing gonad. During normal mammalian development, the tightly regulated spatiotemporal expression of *SRY *within the indifferent genital ridges is required for testis determination to proceed. Deregulation of this expression, either via insufficient *SRY *mRNA concentrations [1,2], delayed *SRY *expression [3], expression of different SRY isoforms [4], and/or the contribution of autosomal loci [5,6], can result in partial or complete failure of testis determination and the formation of ovotestes or ovaries within XY individuals. *SRY *expression is now described within the genital ridges of several eutherian mammals (reviewed in [7]). In the mouse, *Sry *mRNA is first detected by RT-PCR within the somatic cells of the indifferent genital ridges of male embryos at e10.5, with a peak of expression seen at e11.5 followed by a dramatic extinction of expression by e12.5 [8]. These results have been confirmed by *in situ *hybridization [9] and at the protein level [10]. Expression of the human *SRY *transcript is first seen within the indifferent male genital ridge beginning at 41 to 44 days post ovulation, corresponding to Carnagie stages 17–18 [11]. In contrast to the mouse, human *SRY *expression is maintained at low levels within the developing testes for the remainder of gestation, a finding supported by immunohistochemistry results [11]. The genital ridge expression of *SRY *has now been additionally described for the pig, sheep, dog and goat, and collectively reveals the initiation of *SRY *transcription within the indifferent male genital ridge followed by a gradual trailing off of expression more similar to the human model of expression than to the mouse model [12-16].

Although the essential functional role of SRY has been established for some time [17], surprisingly very little is known about the molecular mechanisms that regulate its spatiotemporal expression. The ability of Wilms' tumor 1 (WT1) and the nuclear receptor steroidogenic factor 1 (NR5A1/SF-1/Ad4BP) to bind and transactivate the human or pig *SRY *promoters has been demonstrated [18-21], suggesting that these factors contribute to the tissue-specific expression profile of SRY. Moreover, more recent data have shown that the SRY-related factor SOX9 can also transactivate the pig *SRY *promoter via a consensus SOX9 binding site about 205 bp upstream of the ATG translational start site [22]. After the *SRY *gene is turned on, testis differentiation ensues; Sertoli cells organize into cord structures that encircle immature germ cells termed gonocytes. At the same time, Leydig cells present in the interstitium of the testicular cords differentiate and begin to secrete testosterone. The next stage of male development is sex differentiation, which takes the form of Müllerian duct (female reproductive tract) regression and Wolffian duct (male reproductive tract) development. These events rely on two hormones produced by the fetal testis: testosterone, secreted by Leydig cells, and Müllerian inhibiting substance (AMH/MIS), produced by Sertoli cells. Absence of AMH in humans causes persistent Müllerian duct syndrome, a form of pseudohermaphroditism characterized by the retention of Müllerian duct structures.

The genital ridge expression of *AMH *is described in the human (reviewed in [23]), as well as the mouse [8], pig [14,24,25], sheep [12] and dog [26]. *AMH *expression is restricted to the pre-Sertoli cells of the male genital ridge, always after the initiation of *SRY *expression and commitant to the time of histological testis cord formation. Once *AMH *expression is initiated it continues to be expressed at high levels for the duration of the gestational period. The transcriptional regulation of the *AMH *gene has been studied in detail. Interestingly, several transcription factors involved in primary sex determination (SF-1, WT1, SOX9 and GATA4) are also recruited as important regulators of *AMH *transcription [27-31]. Much like *SRY*, however, our understanding of how these factors work together to direct the sex-specific expression of the *AMH *gene remains incomplete.

A common regulatory factor important for transcription of the *SRY *and *AMH *genes is WT1. WT1 is a zinc finger transcription factor having both DNA and RNA binding properties [32,33]. There are at least 24 different WT1 isoforms due to alternative transcription start sites, RNA editing, and 2 alternative splicing sites. The first alternative splicing site inserts or removes exon 5, which encodes a 17 amino acid segment upstream of the zinc fingers. The second alternative splicing site, at the end of exon 9, inserts or removes three amino acids (KTS) between zinc fingers 3 and 4. Previous studies have shown that WT1 isoforms with or without the KTS tripeptide have distinct biological functions, whereas the alternative splicing site exon 5 has a modulatory role on WT1 function. Although WT1 was first identified as a tumor-suppressant gene of Wilms' tumor [34,35], subsequent studies revealed that WT1 is also essential for embryonic development, especially the kidney and gonads (reviewed in [34,36-38]). In humans, *WT1 *gene mutations are responsible for two urogenital diseases: Denys-Drash syndrome (DDS) and Frasier syndrome. DDS is caused by exonic point mutations in the *WT1 *gene, and is characterized by mesangial sclerosis with early kidney failure, high risk of Wilms' tumor, and varying degrees of gonadal dysgenesis associated with insufficient AMH production [39]. On the other hand, Frasier syndrome, characterized by focal segmental sclerosis, delayed kidney failure and XY gonadal sex reversal, is associated with heterozygous point mutations in intron 9 that cause a shift in the ratio of WT1 isoforms (+/-KTS) towards the -KTS forms [40,41]. The importance of WT1 during gonad development has also be demonstrated by *Wt1*-null mice, which lack kidneys, gonads, and adrenals [42,43]. Moreover, mice lacking the WT1(+KTS) splice variant show complete XY sex reversal and a dramatic reduction in *Sry *expression [44]. Consistent with a role in *SRY *and *AMH *transcription, in vitro studies have shown that WT1(-KTS) can bind and activate the respective gene promoters [19,45,46]. However, since WT1 starts to be expressed in the developing gonadal primordium of the mouse (e9.5–e10.5) somewhat before the *Sry *and *Amh *genes are actually first turned on (e10.5–e12.5) [8,47], and since WT1 is present in several organs where SRY and AMH are not, other factors must cooperate with WT1. Due to its overlapping expression pattern with WT1 in the developing urogenital system [48], GATA4 is an interesting candidate.

GATA4 is a member of the GATA family of zinc-finger transcription factors that recognize the consensus nucleotide sequence WGATAR (called the GATA motif) in the promoter region of target genes. In the mouse, GATA4 is strongly expressed in the somatic cell population of the developing gonad prior to and during the time of sex determination [48]. This expression pattern coincides with the *SRY *and *AMH *genes [8,49], suggesting that GATA4 plays a predominant role in their expression. This hypothesis is supported by *in vivo *experiments, where mice carrying a GATA4 mutation that disrupts its ability to interact with its transcriptional partner FOG2, display abnormal testis development [50]. On a molecular level, this is apparently due to a significant reduction in *Sry *transcription and a block in *Sox9 *and *Amh *expression [50,51]. Thus GATA4, much like WT1, appears to play a predominant role in both primary sex determination and sex differentiation via *SRY *and *AMH *gene regulation. However, since GATA4 is rather broadly expressed, and more specifically, is found in tissues that do not express either *SRY *or *AMH*, it too cannot account by itself for the tight spatiotemporal expression of these genes. Interestingly, the developing gonad is a location where GATA4 and WT1 co-localize with both SRY and AMH [8,47-49]. In this study, we provide the first molecular evidence that a physical and functional cooperation between WT1 and GATA4 contributes to the transcriptional regulation of the *SRY *and *AMH *gene promoters.

## Results

### Species-specific regulation of SRY promoter activity by GATA4

**Figure 1:**
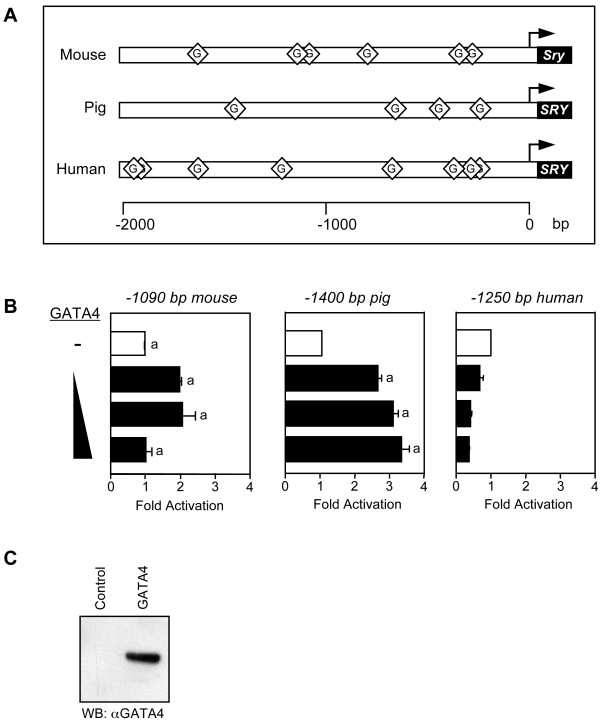
**Transcriptional properties of GATA4 on the mouse, pig, and human *SRY *promoters**. A. Multiple consensus (but not species conserved) GATA binding motifs are present in the first 2 kilobases of the mouse, human, and pig *SRY *promoters; the GATA sites are indicated by the lozenges. B. Ability of GATA4 to transactivate the *SRY *promoter. HeLa cells were co-transfected with either a -1090 bp mouse, -1400 bp pig, or -1250 bp human *SRY*-luciferase promoter construct (500 ng) along with increasing amounts of a GATA4 expression vector (25, 50, or 100 ng). All promoter activities are reported as fold activation over control ± S.E.M. Like letters indicate no statistically significant difference between groups (P > 0.05). C. Western blot analysis of nuclear extracts (10 μg) from HeLa cells overexpressing GATA4.

**Figure 2:**
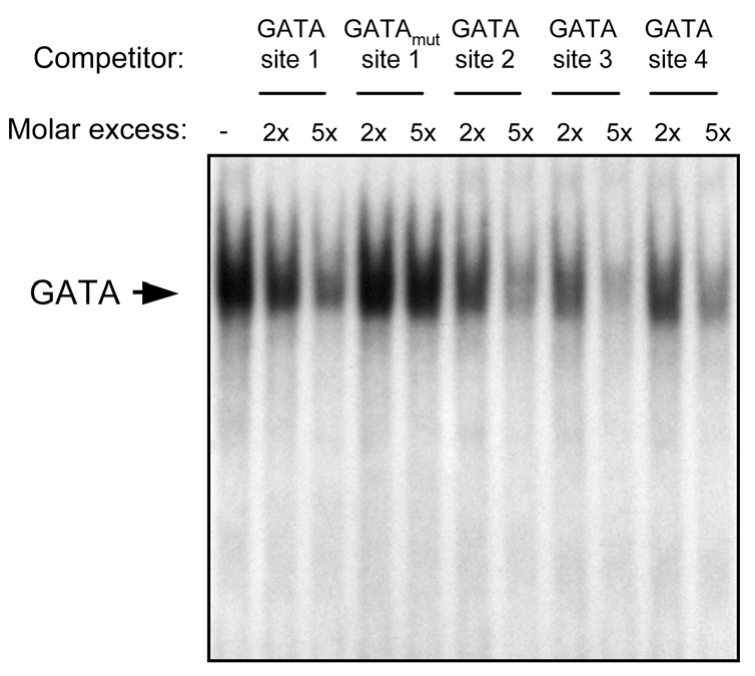
***SRY *promoter GATA motifs bind GATA4**. An EMSA was performed with recombinant GATA4 protein and a ^32^P-labeled oligonucleotide probe corresponding to the first consensus GATA motif (GATA site 1) of the pig *SRY *promoter. Competition with an oligonucleotide containing a mutated GATA motif (GATA to GGTA) was used to confirm the specificity of GATA4 binding (GATA_mut _site 1). GATA4 binding to the more distal GATA motifs (GATA sites 2–4) was then assessed by competition experiments using the indicated oligonucleotides.

There are multiple consensus GATA regulatory motifs within the first 2 kilobases of 5' flanking sequences of the mouse, pig, and human *SRY *genes (Fig. 1A). Although numerous GATA binding sites are present in the different *SRY *promoters, they are not necessarily species conserved which is to be expected as *SRY *5' flanking sequences are generally poorly conserved between mammals [52]. As shown in Fig. 1B, we tested the ability of GATA4, at varying doses, to transactivate the *SRY *promoter from these three species by transient transfection assays in the heterologous HeLa epithelial cell line. HeLa cells do not express GATA4 making it a convenient cell line model for promoter regulation studies involving this factor. Of the three species tested, the pig *SRY *promoter was the most responsive to GATA4 with an activation of about 3-fold (Fig. 1B, middle panel). This was followed by the mouse *Sry *promoter which was activated by 2-fold in the presence of GATA4 (Fig. 1B, left panel). This 2-fold activation approached significance but was not significant (P > 0.05). Interestingly, the human *SRY *promoter was not activated by GATA4 (Fig. 1B, right panel), and this despite the presence of multiple consensus GATA binding motifs and the fact that GATA4 could be strongly overexpressed in HeLa cells (Fig. 1C). Similar results were obtained in other heterologous cell lines such as CV-1 fibroblasts (data not shown). The absence or relatively weak ability of GATA4 to activate the *SRY *promoter suggested that the factor might not bind to *SRY *GATA motifs with high affinity. However, EMSA analysis revealed the contrary. As shown in Fig. 2, GATA4 was able to strongly bind to all four GATA motifs of the proximal pig *SRY *promoter. Similar results were obtained with the GATA binding elements of the mouse and human *SRY *promoters (data not shown). Thus, the mechanism of GATA4 in *SRY *gene regulation is unlikely one of direct transcriptional activation by GATA4 alone.

### Transcriptional cooperation between GATA4 and WT1 on the SRY promoter

**Figure 3:**
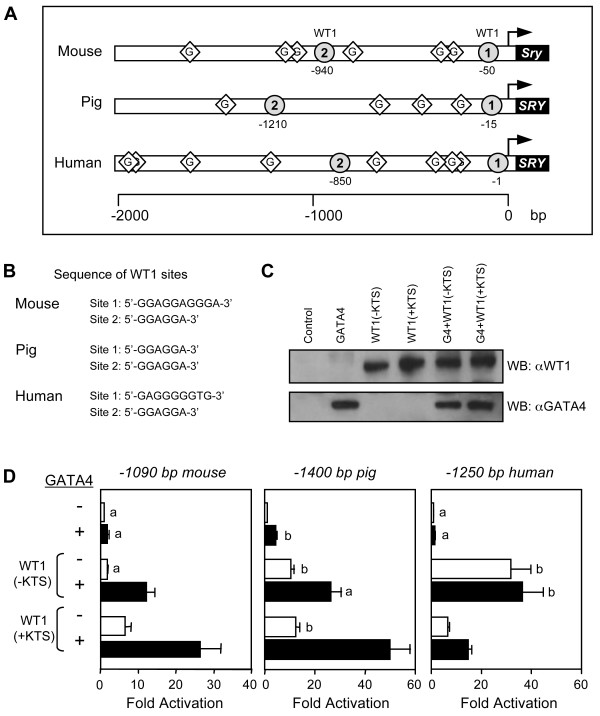
**GATA4 and WT1 transcriptionally cooperate to activate the *SRY *promoter**. A. In addition to multiple GATA motifs, two potential WT1 binding sites (indicated by gray circles) are present in the first 2 kilobases of the mouse, human, and pig *SRY *promoters. B. Nucleotide sequence of the potential WT1 binding sites in the mouse, pig, and human *SRY *promoters. C. Western blot analysis of HeLa cells extracts (10 μg) overexpressing GATA4 and/or WT1 (+/- KTS) isoforms. D. WT1 and GATA4 transcriptionally cooperate. HeLa cells were co-transfected with either a -1090 bp mouse, -1400 bp pig, or -1250 bp human *SRY*-luciferase promoter construct (500 ng) along with an empty vector or expression vectors (500 ng) for WT1(-KTS) or WT1(+KTS) in the absence (-) or presence (+) of GATA4 (50 ng). All promoter activities are reported as fold activation over control ± S.E.M. Like letters indicate no statistically significant difference between groups (P > 0.05).

Although *SRY *5' flanking sequences are known to be divergent in mammals [21,52], in addition to GATA regulatory motifs, 2 partially conserved WT1 binding sites are present within the first 2 kb of 5' flanking sequences of the mouse, pig, and human *SRY *genes (Fig. 3A). The sequences of the putative WT1 binding sites are shown in Fig. 3B. The TCC repeat elements common to the mouse, pig, and human *SRY *sequences have been shown to be recognized by both the -KTS and +KTS isoforms of WT1 [53]. The only exception is the human WT1 binding site 1 (5'-GAGGGGGTG-3') which appears to be preferentially recognized by WT1(-KTS) and not WT1(+KTS) [19]. The WT1 isoforms, much like GATA4, were capable of activating the *SRY *promoter on their own; the magnitude of the activations, however, was greater than GATA4 being on the order of 10–20 fold (Fig. 3D, open bars). Since the WT1 and GATA binding elements lie in close proximity to one another, we tested whether GATA4 could transcriptionally cooperate with WT1 on the *SRY *promoter by performing co-transfection experiments in heterologous HeLa cells (Fig. 3D, black bars). The three species-specific *SRY *promoter constructs used in the co-transfection experiments contained both WT1 binding sites and 4 or more consensus GATA elements. On the mouse *Sry *promoter, both WT1 isoforms strongly synergized with GATA4 with activations reaching 30-fold (Fig. 3D, left panel). Interestingly, the level of GATA4/WT1 synergism was higher with WT1(+KTS) than with WT1(-KTS). Similar results were also observed on the pig *SRY *promoter where activations reached nearly 50-fold (Fig. 3D, middle panel), as well as in other cell lines such as TM4 mouse Sertoli cells (data not shown). In contrast to the mouse and pig, the human *SRY *promoter was strongly activated by WT1(-KTS) alone and synergism with GATA4 only occurred with the WT1(+KTS) isoform (Fig. 3D, right panel). Western blot analysis confirmed the heterologous nature of HeLa cells since they are null for both GATA4 and WT1 (Fig. 3C). It also showed that the GATA4/WT1 synergism was not due to variable expression of either GATA4 or the WT1 isoforms since all proteins (whether expressed individually or in combination) were present at similar levels when overexpressed in HeLa cells (Fig. 3C).

### The proximal WT1 binding site is essential for GATA4/WT1 synergism on the mouse Sry promoter

**Figure 4:**
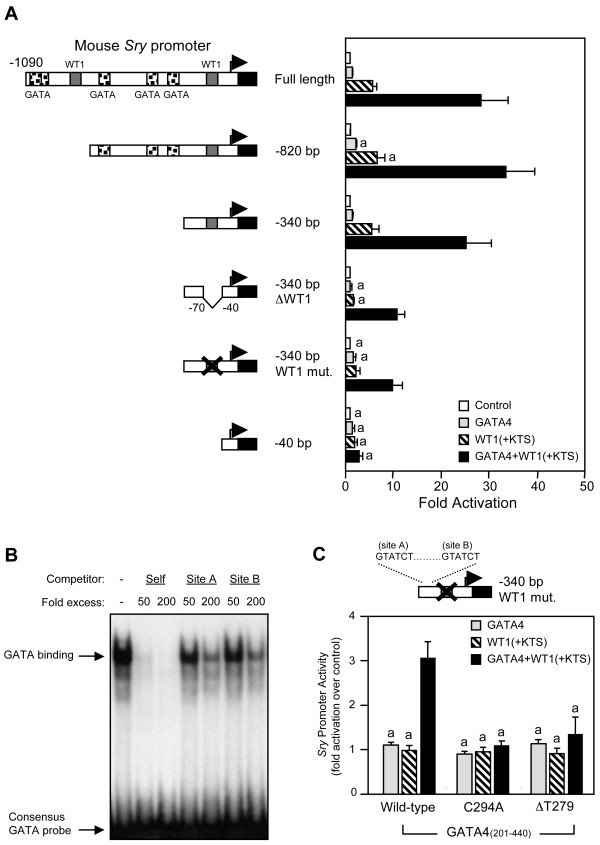
**The mouse *Sry *promoter requires an intact proximal WT1 binding site for full transcriptional synergism by GATA4 and WT1**. A. Deletion and mutation analysis of mouse *Sry *5' flanking sequences in HeLa cells. HeLa cells were co-transfected with the different mouse *Sry *promoters (500 ng) as indicated along with an empty vector (control) or expression vectors for GATA4 and/or WT1(+KTS). B. The mouse *Sry *promoter contains two low affinity GATA binding elements (named sites A and B) located between -340 and -70 bp. An EMSA was performed with recombinant GATA4 protein and a ^32^P-labeled oligonucleotide probe corresponding to the consensus GATA element from the proximal murine *Star *promoter [70]. Competition with unlabeled probe (self) and oligonucleotides corresponding to GATA sites A and B of the mouse *Sry *promoter was used to assess the affinity of GATA4 binding to these sites. C. The low affinity GATA binding sites (A and B) of the proximal mouse *Sry *promoter are functional. The remaining GATA4/WT1 synergism observed on the -340 bp *Sry *construct harboring the WT1 site mutation (-340 bp WT1 mut.) is abolished when two different GATA4 DNA-binding mutants (C294A or ΔT279) are used. For the wild-type and mutated GATA4 constructs, a truncated GATA4 protein (aa 201–440; see diagram in Fig. 6A) was used. For all transfection experiments, promoter activities are reported as fold activation over control ± S.E.M. Like letters indicate no statistically significant difference between groups (P > 0.05).

To assess the binding site requirements for transcriptional synergism between GATA4 and WT1, mouse *Sry *promoter deletion and mutation constructs were prepared and used in co-transfection assays (Fig. 4A). Interestingly, the -340 bp *Sry *promoter construct (containing an intact proximal WT1 site and no consensus GATA sites) was synergistically activated by GATA4 and WT1 to the same extent as the full-length (-1090 bp) *Sry *construct. This suggests that GATA4 binding to its consensus sites on the *Sry *promoter is dispensable for maximal synergism with WT1. Deletion or mutation of the proximal WT1 site, however, abolished the activation by WT1 alone and markedly decreased the synergism between GATA4 and WT1 (Fig. 4A). Thus, in contrast to GATA4, binding of WT1 to its consensus element is critical for GATA4/WT1 synergism on the mouse *Sry *promoter. Interestingly, GATA4/WT1 synergism was not completely abrogated with the WT1 mutant constructs. The remaining synergism is likely due to two low affinity (not perfect consensus) GATA binding motifs present within the -340 to -70 bp sequence since a further deletion to -40 bp eliminated all WT1/GATA4 synergism (Fig. 4A). These two GATA motifs, named sites A (GTATCT) and B (GTATCT), are unique to the mouse *Sry *promoter sequence. Although these sites only weakly bind GATA4 protein (Fig. 4B), they are nonetheless functional as revealed by transfection assay (Fig. 4C). The remaining GATA4/WT1 synergism observed on the -340 bp *Sry *construct harboring the WT1 site mutation (-340 bp WT1 mut.) was abolished when a truncated wild-type GATA4 protein (aa 201–440) was substituted with two GATA4 mutants (C294A or ΔT279) that we have previously shown to be unable to bind to DNA [54]. Thus, GATA4 binding to these two low affinity GATA motifs likely contributes to the observed GATA4/WT1 synergism on the mouse *Sry *promoter. We still cannot rule out the possibility, however, of additional unknown regulatory elements (present between -340 and -70 bp) that might be indirectly activated by GATA4/WT1 overexpression in our heterologous HeLa cell line model.

**Figure 5:**
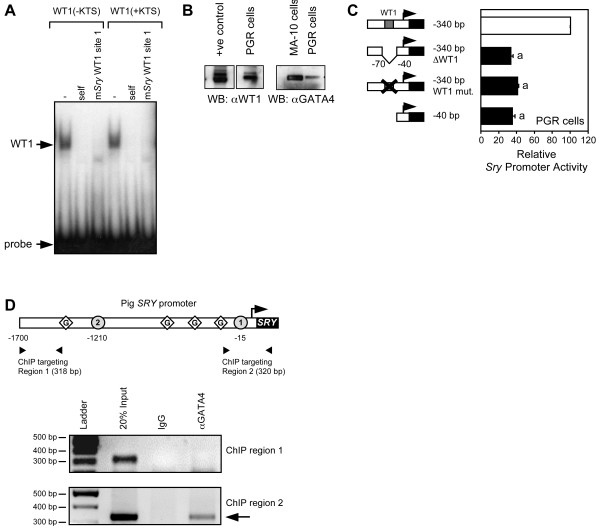
**The proximal WT1 binding site in mouse *Sry *promoter is functional**. A. A DNA-binding assay was performed with in vitro produced WT1 proteins and a ^32^P-labeled oligonucleotide probe corresponding to the consensus WT1 binding site of the human *AMH *promoter. WT1 binding (+ or - KTS isoforms) to the labeled probe was blocked by 10-fold excess of unlabeled probe (self) and unlabeled oligo corresponding to the proximal WT1 site (site 1) of the mouse *Sry *promoter. B. Western blot analysis of nuclear extracts (10 μg) from PGR 9E11 cells (a pig genital ridge cell line) using antisera against WT1 and GATA4. Nuclear extracts from HeLa cells overexpressing WT1(-KTS) and MA-10 Leydig cells were used as positive controls for WT1 and GATA4 expression, respectively. C. Transfection studies performed in homologous PGR cells confirm the importance of the proximal WT1 site for basal *Sry *promoter activity. PGR cells were transfected with the deleted or mutated mouse *Sry *promoter constructs (500 ng) as indicated. Results are shown as % activity relative to the intact -340 bp construct ± S.E.M. Like letters indicate no statistically significant difference between groups (P > 0.05). D. GATA4 is associated with the *SRY *promoter in PGR genital ridge cells. PGR cell lysates were prepared and interaction of GATA4 with the endogenous pig *SRY *promoter was studied by ChIP. An aliquot of chromatin preparation before immunoprecipitation (20% input) was used as positive control. Chromatin was precipitated with a GATA4 antiserum (αGATA4) or incubated with goat-IgG (IgG) which served as a negative control. A 320-bp DNA fragment spanning a portion of the *SRY *promoter containing the proximal WT1 binding site (ChIP targeting region 2) was amplified by PCR as indicated by the arrow. A more distal *SRY *promoter fragment lacking this WT1 site (ChIP targeting region 1) was not amplified.

In a DNA-binding experiment (Fig. 5A), WT1 (+ or - KTS) binding to a known consensus WT1 probe was competed using either unlabeled probe or an oligonucleotide corresponding to the proximal WT1 element (site 1) of the mouse *Sry *promoter. Thus, these results demonstrate that the proximal WT1 site (site 1) is efficiently bound by both WT1 isoforms. The functional importance of the proximal WT1 binding site was confirmed using homologous (WT1 and GATA4-expressing) PGR 9E11 cells, a pig genital ridge cell line [21]. As shown in Fig. 5B, PGR cells express abundant WT1 and GATA4 proteins. Transfection studies carried out in this cell line clearly showed that the deletion or mutation of the proximal WT1 element reduced *Sry *promoter activity to the same level as the minimal promoter (approximately 33% of the intact construct; Fig. 5C). Thus, WT1 binding to its proximal site is crucial for full basal activity of the *Sry *promoter. In our *Sry *promoter deletion experiments (Fig. 4A), we found that GATA binding to its consensus motifs was dispensable for maximal GATA4/WT1 synergism suggesting that GATA4 cooperates with DNA-bound WT1. As shown in Fig. 5D, a ChIP assay using pig PGR cells confirmed that GATA4 is indeed associated with the endogenous *SRY *promoter in close proximity to the proximal WT1 binding site (ChIP targeting region 2). In contrast, a more distal *SRY *promoter fragment lacking the critical WT1 site (ChIP targeting region 1) could not be amplified.

### The zinc finger domains and the C-terminal region of GATA4 are required for the transcriptional cooperation and physical interaction with WT1

**Figure 6:**
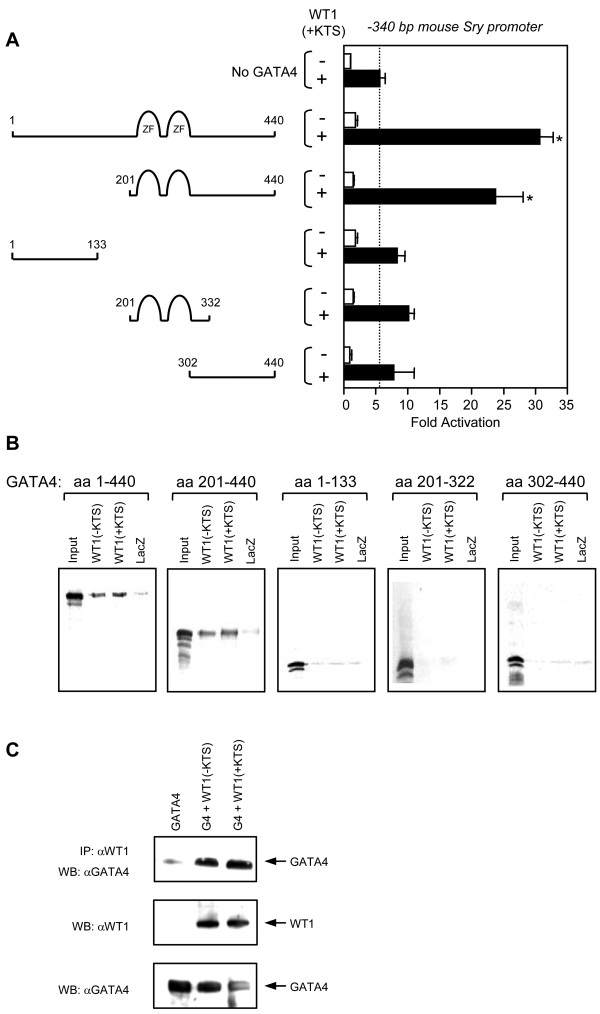
**The zinc finger domains and C-terminal region of the GATA4 protein are required for the transcriptional cooperation and direct interaction with WT1**. A. The domain of the GATA4 protein involved in the transcriptional cooperation with WT1 was identified using the full-length GATA protein and four different GATA4 deletion mutants as indicated. HeLa cells were co-transfected with the -340 bp mouse *Sry *promoter (500 ng) along with an empty vector or expression vectors for the different GATA4 constructs in the absence (-) or presence (+) of a WT1(+KTS) expression vector (500 ng). All promoter activities are reported as fold activation over control ± S.E.M. The dotted line indicates the activation induced by WT1(+KTS) alone. *, Significantly different from the activation elicited by WT1 alone (P < 0.05). B. In vitro pull-down assays were performed using 500 ng of bacterially produced HIS-WT1(-KTS), HIS-WT1(+KTS) and HIS-LacZ fusion (-ve control) proteins and in vitro translated ^35^S-labeled GATA4 proteins. After extensive washes, the bound proteins were separated on a 10% SDS-PAGE gel and visualized by autoradiography. Input corresponds to 10% of the total ^35^S-GATA4 used in each assay. C. GATA4 interacts with both WT1(-KTS) and WT1(+KTS) isoforms. HeLa cells were transfected with expression vectors for either GATA4 alone or GATA4 in the presence of WT1 (+ or -KTS isoforms). A 100-μg aliquot of nuclear extract was then immunoprecipitated (IP) with an antibody for WT1. The precipitated material was then subjected to Western blot (WB) analysis for GATA4. The nuclear extracts used in the IPs were also directly immunoblotted (2.5 μg per lane) to control for the specificity of the GATA4 and WT1 antisera.

The strong transcriptional cooperation between GATA4 and WT1 on the *SRY *promoter suggests that both factors contact each other through a direct protein-protein interaction. The essential nature of this interaction is all the more evident given that GATA4 binding to its consensus motifs is not required for maximal synergism with WT1 (Fig. 4A). To map the domains of the GATA4 protein that interact with WT1 and are important for synergism on the *Sry *promoter, a series of truncated GATA4 proteins were constructed as depicted in Fig. 6A. The GATA4 protein contains two independent activation domains that flank its DNA-binding domain. Deletion of the N-terminal region (aa 1–200) had no significant effect on the transcriptional synergism between GATA4 and WT1(+KTS) (Fig. 6A). However, GATA4/WT1 synergism was lost using proteins consisting solely of the N-terminal region (aa 1–133), zinc finger region (aa 201–322) or C-terminal domain (aa 302–440). Thus, the zinc finger and C-terminal domains of the GATA4 protein are both required for its ability to transcriptionally cooperate with WT1. Next, the domain of the GATA4 protein involved in the direct physical interaction with WT1 was mapped using in vitro pull-down assays (Fig. 6B). Consistent with the transcription data (Fig. 6A), the WT1 protein (- or +KTS) could physically interact with the intact (wild-type) GATA4 protein (Fig. 6B, far left panel) but not with mutant GATA4 proteins that removed the zinc fingers or C-terminal portions of the protein (Fig. 6B, 3 rightmost panels). Thus, the zinc finger and C-terminal domains of GATA4 are also essential for the physical interaction with WT1. The ability of GATA4 to interact with WT1 in the absence of DNA-binding was further confirmed by a co-immunoprecipitation experiment (Fig. 6C).

### GATA4/WT1 transcriptional synergism also regulates activity of the AMH promoter

**Figure 7:**
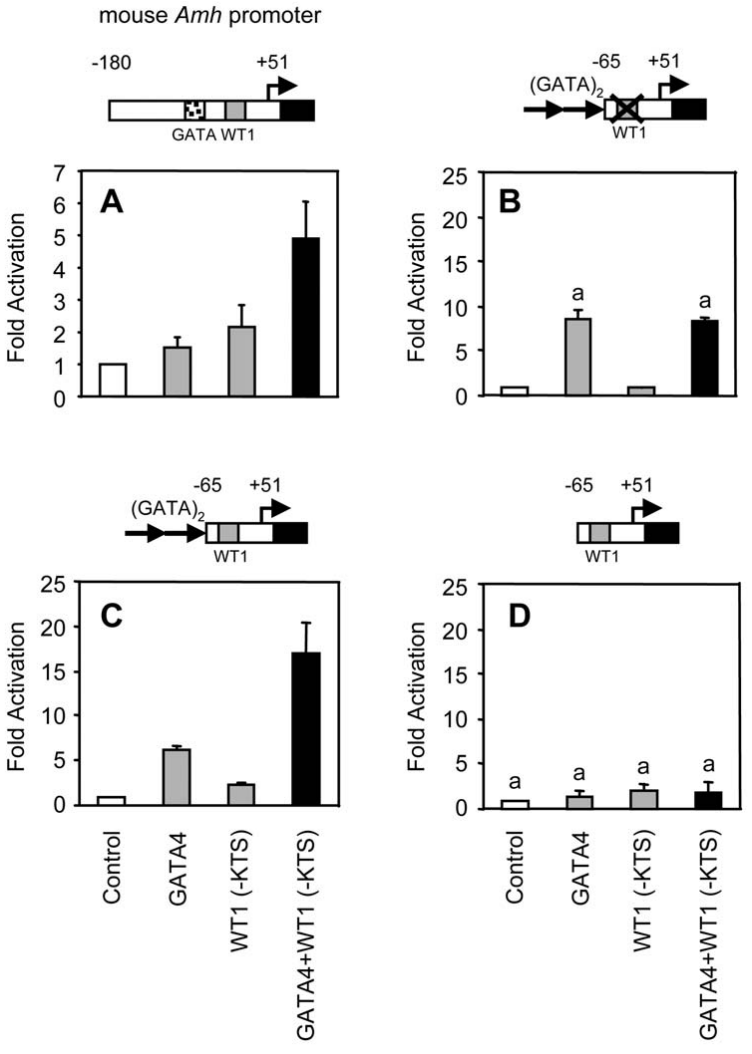
**Transcriptional synergism between GATA4 and WT1 on the *Amh *promoter requires GATA4 and WT1 binding to their respective regulatory elements**. A. The native -180 bp *Amh *promoter containing the GATA and WT1 elements in their natural context. B. A synthetic reporter containing two consensus GATA motifs upstream of the minimal *Amh *promoter with its WT1 binding site mutated. C. A synthetic reporter containing two consensus GATA motifs upstream of the minimal *Amh *promoter with an intact WT1 binding site. D. The minimal *Amh *promoter containing only a WT1 binding site. In all experiments, HeLa cells were co-transfected with the different promoter constructs (500 ng) along with an empty vector or expression vectors for the WT1(-KTS) (500 ng) and GATA4 (100 ng) used alone or in combination. All promoter activities are reported as fold activation over control ± S.E.M. Like letters indicate no statistically significant difference between groups (P > 0.05).

Since we found in the present study that GATA4 and WT1 could cooperate to regulate transcription from the *SRY *promoter, we surmised that the expression of other gonadal genes might also be modulated by this functional cooperation. Another obvious target in the sex determination/sex differentiation pathway is the *AMH *gene since its proximal promoter region contains species-conserved binding elements for both GATA4 and WT1 [see Additional file 1]. Both factors are known regulators of *AMH *transcription [45,48], which is mediated through their direct association with the proximal *AMH *promoter as shown by ChIP (ref. [45] and data not shown). To better define the role of GATA4 as a transcriptional partner for WT1 in the cell-specific and developmental regulation of the *AMH *gene, we tested whether GATA4 and WT1 could synergistically activate the *AMH *promoter. In our co-transfection experiments, we used the mouse -180 bp A*mh *promoter which contains the GATA and WT1 binding sites in their normal context, as well a series of synthetic constructs containing either GATA and/or WT1 elements placed upstream of the minimal *Amh *promoter (Fig. 7). Although crucial for *Amh *transcription *in vivo *[50], GATA4 by itself is a poor activator of the *Amh *promoter (Fig. 7A). While WT1 alone weakly activated the *Amh *promoter, and in contrast to the *Sry *promoter, only the -KTS isoform was transcriptionally active. In the presence of both proteins, however, a strong synergistic activation was observed not only on the native -180 bp *Amh *promoter but also a synthetic reporter consisting of two consensus GATA motifs placed upstream of the minimal (-65 bp) *Amh *promoter containing an intact WT1 binding element (Fig. 7A and 7C). Finally, to assess the binding site requirements for GATA4/WT1 synergism, mutations or deletions of the *Amh *promoter were generated (Fig. 7B and 7D). Although activation by GATA4 alone was retained using a construct with intact GATA motifs but a mutated WT1 element, GATA4/WT1 synergism was lost (Fig. 7B). Similarly, no GATA4/WT1 synergism was observed on a construct containing an intact WT1 binding site but deleted of the GATA motifs (Fig. 7D). Thus, again unlike the *SRY *promoter, maximal GATA4/WT1 synergism on the *AMH *promoter requires both GATA4 and WT1 binding.

### A mutated form of WT1 (WT1 R394W) that causes the retention of Müllerian ducts in humans fails to synergize with GATA4

**Figure 8:**
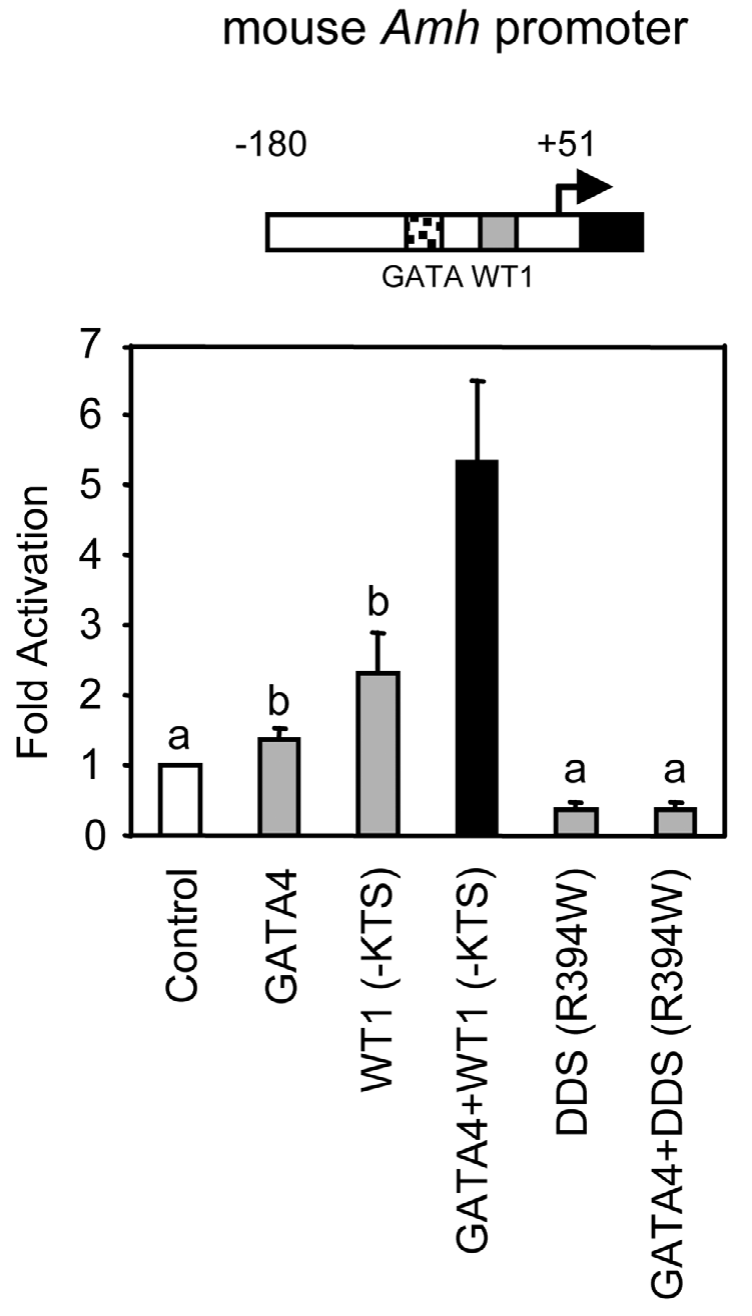
**A mutated form of WT1 (WT1 R394W) that causes retention of Müllerian ducts in humans fails to synergize with GATA4**. A. HeLa cells were co-transfected with the mouse -180 bp *Amh *promoter and either an empty expression vector (control) or expression vectors for GATA4 (100 ng), WT1(-KTS) wild-type (500 ng) or WT1(-KTS) R394W DDS mutant (500 ng), used alone or in combination as indicated. All promoter activities are reported as fold activation over control ± S.E.M. Like letters indicate no statistically significant difference between groups (P > 0.05).

There are several naturally occurring WT1 mutants known to cause male pseudohermaphroditism with retention of Müllerian ducts (reviewed in [55]). One of these mutants, WT1 R394W, was shown to be a poor activator of the *AMH *promoter [45]. To better explain the molecular mechanism behind the human phenotype associated with the mutation, we were interested in verifying whether the WT1 R394W mutant could still transcriptionally cooperate with GATA4. As shown in Fig. 8, the WT1 R394W mutant, unlike its wild-type counterpart (- or + WT1 isoforms), not only failed to activate the *Amh *promoter but also failed to synergize with GATA4. This lack of synergism was not related to the stability or expression level of the mutant protein (data not shown). Rather, the lack of synergism is most likely attributable to a decrease in DNA binding affinity of the mutant WT1 protein for its binding element [45]. This result therefore supports our previous observation that GATA4/WT1 synergism on the *AMH *promoter absolutely requires binding of both factors to their respective regulatory elements (Fig. 7). Thus, a failed transcriptional synergism between GATA4 and the mutant WT1 protein at the level of the *AMH *promoter could be a contributing factor to the improper male sex differentiation seen in WT1 R394W patients involving insufficient AMH production.

## Discussion

SRY is the genetic trigger for male sex determination in mammals. Although its critical developmental function has been established for more than 15 years now [17], the molecular mechanisms that regulate its tight spatiotemporal expression in the developing genital ridge have remained elusive. Although a number of transcriptional regulators including SF-1, SOX9 and WT1 have been proposed to participate in *SRY *gene regulation [18-21,46], the sum of these factors still do not explain its highly restricted expression pattern. We report here, a novel transcriptional cooperation between WT1 and the GATA4 transcription factor. This important cooperation not only markedly enhances transcription from the *SRY *promoter but also targets the promoter of the *AMH *gene involved in male sex differentiation. Thus, our data provide new insights into the molecular mechanisms that control the tissue- and developmental-specific expression of these two critical genes and identify GATA4 as a potential causative factor in cases of abnormal human sex development such as Frasier syndrome (XY male-to-female sex reversal with intact *SRY *gene) and DDS syndrome (incomplete sex differentiation associated with reduced AMH levels).

In the mouse, GATA4 is strongly expressed in the somatic cell population of the developing gonad prior to and during the time of *Sry *expression [48]; GATA4 is also detected in Sertoli cells of the early fetal testis in humans [56]. Moreover, more recent knockout data in the mouse revealed that a functional GATA4 protein is required for *Sry *expression and hence normal testis cord formation [50], suggesting that GATA4 is essential for either the onset or up-regulation of *Sry *transcription in the developing male genital ridge. Although the *SRY *promoters from mouse, pig and human all contain multiple consensus GATA regulatory motifs, only the mouse and especially the pig *SRY *5' flanking sequences were activated by GATA4 (Fig. 1). This was not surprising knowing that while SRY function is highly conserved across all mammalian species, *SRY *promoter regulatory sequences are not. Thus, it is entirely conceivable that GATA4 might directly regulate *SRY *transcription in some mammalian species (such as the pig and mouse) but not others (such as the human). For these other species, GATA4 likely contributes to *SRY *transcription through interactions with other transcriptional regulators. Due to its implication in many clinical cases of abnormal human sex development, an interesting candidate for this GATA4-interacting factor is WT1. Indeed, a previous study using transgenic mice expressing green fluorescent protein (GFP) under the control of the *Sry *promoter has shown that *Sry *is expressed only in somatic cells of the mouse genital ridge that also co-express GATA4 and WT1 [57]. Consistent with its role in *SRY *gene regulation, WT1 has been reported to activate the *SRY *promoter [19,46]. Much like GATA4, however, it alone cannot account for the highly restricted *SRY *expression pattern since WT1 is found in tissues where SRY is not. Given the co-localization of GATA4 and WT1 in the genital ridge, we surmised that both factors might cooperate to regulate the *SRY *gene. Indeed, GATA4 strongly synergized with WT1 to activate the *SRY *promoter of all three species tested (Fig. 3D). Thus, we propose that *SRY *expression is controlled by a GATA4/WT1 transcriptional cooperation rather than by the action of GATA4 or WT1 alone. Although this mechanism helps us to better understand the proper spatiotemporal expression of the *SRY *gene, other factors must also participate. This includes SOX9, SF-1, and especially FOG2. *Fog2*-null mice have disrupted testicular development and markedly reduced *Sry *expression [50]. FOG2 was originally cloned as a cofactor for GATA4 and this interaction is essential for normal testis differentiation [50,51]. Although most in vitro studies point to FOG2 acting as a co-repressor for GATA4 transcriptional activity [58], whether this is actually the case in vivo remains to be shown.

Our results provide strong evidence that both the WT1(-KTS) and WT1(+KTS) isoforms act as transcriptional activators of the *SRY *promoter. It is generally accepted, however, that WT1(-KTS) functions as a transcriptional regulator whereas WT1(+KTS) plays a role in other cellular processes, such as RNA processing [59]. Indeed, to date, only a few genes have been identified as potential transcriptional targets of WT1(+KTS). For example, transactivation of the *CDH1 *(cadherin 1/E-cadherin) gene, which encodes a calcium-dependent cell adhesion protein, is enhanced by WT1 (+KTS) through GC-rich and CAAT box sequences [60]. Moreover, WT1(+KTS) can activate the promoter of the human *MYC *(*C-MYC*) gene via two WT1 binding sites [61]. We found that the mouse *Sry *promoter contains two possible WT1 binding sites, a distal site at -940 bp (5'-GGAGGA-3') and a proximal one at -50 bp (5'-GGAGGAGGGA-3') upstream of the transcription start site. Their sequences are antisense to a TCC-repeat element which has been identified as a binding motif for both WT1(-KTS) and WT1(+KTS) [53]. Indeed, our results showed that WT1(+KTS) binds to the proximal site (Fig. 5A) and activates mouse *Sry *promoter activity alone and in association with GATA4 (Fig. 3D). Thus taken together, although the target genes of WT1(+KTS) are limited in comparison to WT1(-KTS), WT1(+KTS) nonetheless appears to be an important transcriptional regulator of the *SRY *gene. This is not surprising given that the best known human *WT1 *mutation associated with sex reversal (Frasier syndrome) produces a shift in the WT1 +/-KTS ratio that favors the -KTS isoform [40,41].

Interestingly, the level of GATA4/WT1 synergism on the *SRY *promoter was much stronger when the WT1(+KTS) isoform was used. Importantly, this observation might improve our understanding of *Sry *regulation in mutant mice as reported by Hammes et al. [44]. They showed that mutant mice that express only WT1(-KTS) or WT1(+KTS) have distinct functions in sex determination and gonad development [44]. The mouse mutant lacking the WT1(+KTS) isoform exhibited a reduction in *Sry *expression (less than 25% of wild-type) leading to a block in testis development. In contrast, the mouse mutant lacking WT1(-KTS) still showed modest expression of male specific genes (*Sox9 *and *Amh*) downstream of *Sry*, suggesting that WT1(+KTS), and not WT1(-KTS) is essential for *Sry *expression and consequently normal testis development. We show here that the level of GATA4/WT1(+KTS) synergism on the mouse *Sry *promoter was more than twice that of GATA4/WT1(-KTS). Thus, our findings suggest that the reduction in *Sry *expression seen in the mouse lacking WT1(+KTS) might be the result of a lack of appropriate GATA4/WT1(+KTS) synergism and hence, insufficient activation of the *Sry *promoter. The importance of WT1 in male sex determination has also been well-documented in the human since several diseases have been shown to be associated with *WT1 *mutations, such as DDS and Frasier syndrome. Male-to-female sex reversal seen in patients with Frasier syndrome is caused by heterozygous mutations in exon 9 of *WT1*, which as previously mentioned, results in reduced levels of the WT1(+KTS) isoform and an concomitant increase in WT1(-KTS). Thus, WT1(+KTS) is believed to be critical for testis determination in the human as in the mouse. Contrary to these in vivo data, however, previous reports [19,20], as well as our present in vitro studies show that the human *SRY *promoter to be comparatively less responsive to WT1(+KTS) than WT1(-KTS). Despite this observation, we found that GATA4 enhances WT1(+KTS) action on the human *SRY *promoter; an effect that was not observed with the WT1(-KTS) isoform (Fig. 3D), suggesting that WT1(+KTS) also likely contributes to human *SRY *transcription in cooperation with GATA4.

In the mouse, immediately after sex determination (testis differentiation), male sex differentiation ensues with the onset of *Amh *expression by the newly differentiated Sertoli cells. To date, four transcription factors, all acting within the first 180 bp of 5' flanking sequences, have been proposed to be involved in the sex-specific regulation of the *Amh *gene: SF-1, WT1, SOX9, and GATA4 [27-31]. Based on several in vitro and in vivo studies [27-29], up-regulation of *Amh *gene expression in the fetal testis appears to require the presence of both SF-1 and WT1. Moreover, it has been reported that SOX9 binding to its specific response element is essential for the initiation of *Amh *transcription [27]. Studies from our group and others have also shown GATA4 to be an important player in *AMH *gene regulation [54,62,63]. We now show that the WT1-dependent regulation of the *Amh *promoter, much like the *Sry *promoter, is markedly enhanced by GATA4. An important difference, however, was the promoter-specific response to the WT1 isoforms. Consistent with previous reports [29], WT1(-KTS) was the transcriptionally active isoform on the *Amh *promoter (Fig. 7 and data not shown). Thus, WT1(+KTS) and WT1(-KTS) appear to have different actions on the *SRY *and *AMH *promoters as has been described for other WT1 target genes involved in the sex determination pathway [19,20,45,64].

## Conclusion

Taken together, our data not only provide new insights into the molecular mechanisms that contribute to the tissue-specific expression of the *SRY *and *MIS *genes but also highlight the importance of GATA4 a key regulator of gonadal development by acting a two levels in the mammalian sex determination and differentiation cascade.

## Methods

### Plasmids

The mouse, pig and human *SRY *promoters were amplified by PCR using the corresponding species-specific genomic DNA as template and the following pairs of primers: mouse (forward: 5'-CGGGATCCGCTGTATTGTCAATAAAACAG-3', reverse: 5'-GGGGTACCGACAATTGTCACCAGTCCC-3'); pig (forward: 5'-CGGGATCCTTTGAGTTCCAAGG-3', reverse: 5'-GGGGTACCGAAAAGGGGGAGGAAGCG-3'); human (forward: 5'-CGGGATCCAATTCATATAGCTTTTTGTGTCC-3', reverse: 5'-GGGGTACCTCAACACCCCCTCAAC-3'). The different promoters fragments were subsequently cloned into the *Bam*HI/*Kpn*I site of a modified luciferase expression vector [65]. Deletion constructs for the mouse *Sry *promoter were generated by PCR using the following pairs of primers: -820 bp (forward: 5'-CGGGATCCGCCGTAGTAGACTATGATAC-3', reverse: 5'-GGGGTACCGACAATTGTCACCAGTCCC-3'); -340 bp (forward: 5'-CGGGATCCCTTTCCACTACTTTTGCA-3', reverse: 5'-GGGGTACCGACAATTGTCACCAGTCCC-3'); -40 bp (forward: 5'-CGGGATCCTTACACACGTTAAATATTAAAATC-3', reverse: 5'-GGGGTACCGACAATTGTCACCAGTCCC-3'). Promoter fragments were then cloned into the luciferase reporter as described above. The -340 mouse *Sry *promoter construct with a specific deletion of the WT1 response element (ΔWT1) was achieved by cloning a -340 to -70 bp *Sry *promoter fragment, obtained by PCR (forward primer: 5'-CGGGATCCCTTTCCACTACTTTTGCA-3', reverse primer: 5'-GAAGATCTCTAGTCCAGCCCAACTAATC-3'), in front of the minimal mouse *Sry *promoter construct. A -340 bp mouse *Sry *promoter construct harboring a mutation that inactivates the WT1 element (GGAGGAGGGA to GGTGTTGGGT) was generated using the QuikChange XL mutagenesis kit (Stratagene, La Jolla, CA) according to the manufacture's recommendations along with the following oligonucleotides – sense: 5'-GACTAGGGAGGTCCTGAAGGTGTTGGGTTAAATATTTTCTTACAC-3'; antisense: 5'-GTGTAAGAAAATATTTAACCCAACACCTTCAGGACCTCCCTAGTC-3'. The natural and synthetic murine *Amh*-luciferase promoter constructs have been described previously [31]. The synthetic *Amh *promoter construct containing a WT1 binding site mutation was generated by site-directed mutagenesis using the following primer pair – sense: 5'-TACAGCAAGGCCCGGGCGGCCCCGCTTATATGTA-3'; antisense: 5'-TACATATAAGCGGGGCCGCCCGGGCCTTGCTGTA-3'. Finally, the WT1(-KTS) DDS (R394W) mutant was also made by site-directed mutagenesis using the wild-type WT1 cDNA as template and the following primers – sense: 5'-CTTCAGATGGTCGGACCAGGAAAACTTTCGCTG -3'; antisense: 5'-CAGCGAAAGTTTTCCTGGTCCGACCATCTGAAG-3'. The GATA4 expression vectors (wild-type, deletions and DNA-binding mutants) have been described previously [31,54,62,66]. Expression vectors for the wild-type mouse WT1 isoforms were kindly provided by Dr. Jerry Pelletier (McGill University). All plasmid constructs were verified by sequencing.

### Cell culture and transfections

Pig genital ridge PGR 9E11 cells [21] were grown in DMEM supplemented with 10% newborn calf serum at 37°C and 5% CO2. The human cervical carcinoma HeLa cell line was maintained in 1:1 DMEM/Ham's F-12 containing 10% fetal bovine serum. HeLa cells were transfected in 24-well culture plates using the calcium phosphate precipitation method [67]. PGR 9E11 cells were seeded in 12-well plates and transfected using the LipofectAMINE reagent (Invitrogen, Burlington, Canada). In brief, 2.5 μg of *SRY *promoter-luciferase reporters were transfected with 2.5 μl of LipofectAMINE reagent per well in serum- and antibiotic-free media. A renilla (phRL-TK) luciferase plasmid (Promega, Madison, WI) was used as an internal control. Complete medium was added 5 h after transfection. The next day, the cells were harvested and analyzed using the Dual-Luciferase reporter assay system from Promega. The data reported represent the averages of at least three experiments, each done in duplicate. For the experiments presented herein, the internal control was unaffected by either GATA4 or WT1 overexpression [see Additional file 2].

### Nuclear extracts and Western blots

Nuclear extracts were prepared by the procedure outlined by Schreiber et al. [68]. In Western analyses, 10-μg aliquots of nuclear extracts from untreated PGR 9E11 or transfected HeLa cells with GATA4 and/or WT1 expression vectors were separated by SDS-PAGE and then electrotransferred to Hybond PVDF membranes (GE Healthcare Life Sciences, Baie d'Urfé, Canada). Immunodetection of the GATA4 protein was achieved using a GATA4 antiserum (catalog # sc-1237; Santa Cruz Biotechnology, Santa Cruz, CA). WT1 proteins were detected using an anti-WT1 antibody (catalog # sc-192; Santa Cruz) and a VECTASTAIN-ABC-Amp Western blot detection kit (Vector Laboratories Canada, Burlington, Canada). Duolux (Vector) was used as chemiluminescent substrate.

### DNA binding (EMSA) assays

To assess binding of GATA4 to the consensus GATA sites in the pig *SRY *promoter, in vitro translated GATA4 protein was prepared using the TNT system from Promega. DNA binding assays were performed using a ^32^P-labeled double-stranded oligonucleotide corresponding to the proximal GATA site (GATA site 1) of the pig *SRY *promoter – sense oligo: 5'-GATCCGCCTTATTATCATAATAAA-3'; antisense oligo: 5'-GATCTTTATTATGATAATAAGGCG-3'. Competition with an oligonucleotide containing a mutated GATA motif (GATA to GGTA) was used to confirm the specificity of GATA4 binding – GATA_mut _site 1 (sense: 5'-GATCCGCCTTATTACCATAATAAA-3', antisense: 5'-GATCTTTATTATGGTAATAAGGCG-3'). Competition experiments using a series of double-stranded oligonucleotides were then used to confirm GATA4 binding to the more distal GATA motifs in the pig *SRY *promoter – GATA site 2 (sense: 5'-GATCCATTGGGTTATCTTGAATCA-3', antisense: 5'-GATCTGATTCATGATAACCCAATG-3'); GATA site 3 (sense: 5'-GATCCACATACTGATAATCATCAA-3', antisense: 5'-GATCTTGATGATTATCAGTATGTG-3'); GATA site 4 (sense: 5'-GATCCCCAAGGTTATCTGTTTTTA-3', antisense: 5'-GATCTAAAAACAGATAACCTTGGG-3'). Recombinant GATA4 protein was also used to assess the affinity of GATA4 binding to two non-consensus GATA motifs (named sites A and B) in the proximal mouse *Sry *promoter. For this specific experiment, the consensus GATA element of the proximal murine *Star *promoter (sense: 5'-GATCCACTTTTTTATCTCAAGTGA-3'; antisense: 5'-GATCTCACTTGAGATAAAAAAGTG-3') was used as labeled probe. Competition using oligos corresponding to sites A (sense: 5'-GTTCTTTGTATCTTAATACT-3'; antisense: 5'-AGTATTAAGATACAAAGAAC-3') and B (sense: 5'-CTTGACAGTATCTAGGTTCA-3'; antisense: 5'-TGAACCTAGATACTGTCAAG-3') was used to assess the affinity of GATA4 binding to these two sites. A similar approach was used to demonstrate WT1 binding to the proximal WT1 element in the mouse *Sry *promoter. In vitro translated WT1(-KTS) and WT1(+KTS) proteins were prepared using the TNT system (Promega). WT1 binding assays were performed using a ^32^P-labeled double-stranded oligonucleotide corresponding to the distal WT1 element of the human *AMH *promoter (sense oligo: 5'-GGATCACTGGGGAGGGAGATAGGA-3', antisense oligo: 5'-GATCTCCTATCTCCCTCCCCAGTG-3') which has been described as a consensus binding site for both the -KTS and +KTS isoforms of WT1 [20]. Competition experiments using a double-stranded oligonucleotide corresponding to the proximal WT1 site in mouse *Sry *promoter (sense oligo: 5'-GGATCCTGAAGGAGGAGGGATAA-3', antisense oligo: 5'-GATCTTATCCCTCCTCCTTCAGG-3') was used to confirm WT1 binding to the *Sry *promoter. For all EMSA experiments, binding reactions and electrophoresis conditions were as described previously [48].

### Production of HIS fusion proteins

Recombinant histidine-tagged WT1 fusion proteins (HIS-WT1) were obtained by cloning the coding region of the mouse WT1 protein in-frame with HIS using the commercially available pRSETB fusion protein vector from Invitrogen. The resulting construct was introduced into the Escherichia coli strain BL21, and the fusion protein was produced by inducing the bacterial culture with IPTG. After induction, the bacterial culture was lysed by sonication and the fusion protein was purified using a TALON metal affinity resin (BD Biosciences, Mississauga, Canada) according to instructions outlined by the manufacturer.

### In vitro pull-down assays

In vitro pull-down (protein-protein interaction) assays were done using ^35^S-labeled in vitro translated GATA4 proteins (wild-type and deletion mutants) and the purified HIS-WT1 and HIS-LacZ fusion proteins coupled to TALON metal affinity resin. The ^35^S-labeled GATA4 proteins were obtained using the TNT system as described above; the amino acid positions of the different GATA4 proteins used are given in Fig. 6A. Proteins were incubated in 500 μl binding buffer (20 mM Hepes pH 7.9, 70 mM KCl, 20% Glycerol, 0.5% Triton X-100) supplemented with 0.01% BSA for 30 min at room temperature. Bound immunocomplexes were washed three times in washing buffer (60 mM NaH2PO4, 600 mM NaCl, 20% Glycerol, 0.75% Triton X-100), resuspended in Laemmli buffer, and subjected to SDS-PAGE. Proteins were finally electrotransferred to Hybond PVDF membrane and visualized by autoradiography.

### Co-immunoprecipitation

Nuclear extracts were prepared from HeLa cells transfected with expression vectors for GATA4 or a combination of GATA4 and WT1. A 100-μg aliquot of each extract was then immunoprecipitated overnight using the WT1 antibody in binding buffer as described previously [69]. Immunocomplexes were then collected by incubation with 20 μl of protein G-Sepharose beads (GE Healthcare Life Sciences) at 4°C for 1 h. Bound immunocomplexes were washed four times with binding buffer, resuspended in 30 μl of 1× Laemmli buffer, and subjected to SDS-PAGE. Proteins were electrotransferred to PVDF membrane and subjected to immunoblotting using a 1:1000 dilution of the GATA4 antibody and detected with the Vectastain-ABC-Amp Western blot detection kit.

### Chromatin immunoprecipitation (ChIP) assay

PGR cells (4 × 10^7^) were cross-linked with 1% formaldehyde (final concentration) for 10 min at 37°C. The cells were then washed 3 times with PBS, resuspended in ChIP lysis buffer [1% SDS, 10 mM EDTA, and 50 mM Tris-HCl (pH 8.0)], and incubated for 10 min at 4°C. The lysed cells were sonicated on ice using a BRANSON 450 Sonicator for 5 cycles of 30-sec pulses at 3.5 output control and 80% duty cycle in order to obtain DNA fragments between 200 bp and 1000 bp in size. After centrifugation of the solution for 10 min at 4°C, the supernatant was precleared for 2 h at 4°C with 20 μl of a protein G-Sepharose bead slurry (GE Healthcare Life Sciences) that had been prepared by washing it 3 times with RIPA buffer [50 mM Tris-HCl (pH 8.0), 5 mM EDTA, 150 mM NaCl, 0.1% SDS, 0.5% Igepal CA-630 (Sigma-Aldrich Canada, Oakville Canada)]. After centrifugation for 10 min at 4°C, 500 μl of supernatant was subjected to overnight incubation at 4°C with 0.4 μg anti-GATA4 or control goat IgG (Santa Cruz) in immunoprecipitation buffer [20 mM Tris-HCl (pH 8.0), 2 mM EDTA, 150 mM NaCl, 1% Triton X-100]. The immunocomplexes were recovered by a 2 h incubation with 20 μl protein G Sepharose beads. The precipitates were washed 5 times with 1 ml RIPA buffer, 5 times with 1 ml high salt RIPA buffer [50 mM Tris-HCl (pH 8.0), 5 mM EDTA, 500 mM NaCl, 0.1% SDS, 0.5% Igepal CA-630], 3 times with LiCl buffer (250 mM LiCl and 0.1% Igepal CA-630), and finally twice with 1 ml of TE buffer [10 mM Tris-HCl (pH 8.0), 1 mM EDTA]. Protein-DNA complexes were eluted from protein G Sepharose beads by addition of elution buffer (1% SDS, 0.1 M NaHCO_3_) and rotation at room temperature for 15 min. Cross-links were reversed by addition of 200 mM of NaCl and heating at 65°C for 4 h. Proteins were degraded using proteinase K treatment (1 h at 55°C) and the DNA fragments were purified using a QIAquick gel extraction kit (Qiagen, Mississauga, Canada). PCR amplifications were done using 1 μl of input chromatin sample and 5 μl of GATA4-immunoprecipitated DNA sample with primers specific for either the distal (ChIP region 1) pig *SRY *promoter (forward primer: 5'-GTATTCACTTATTTCATTTGGTAAGCCA-3'; reverse primer: 5'-CGAGGTGAACATAACACTTC-3') or the proximal (ChIP region 2) pig *SRY *promoter (forward primer: 5'-TACTGGGGGCGGAGAAATTG-3'; reverse primer: 5'-GGGTCGCTTGACACGATCCT-3'). PCR amplifications were carried out at 94°C for 5 min, followed by 40 cycles of 94°C for 30 sec, 60°C for 30 sec, and 72°C for 30 sec, and a final extension of 5 min at 72°C. The PCR products were analyzed by electrophoresis on a 2% ethidium bromide-stained agarose gel. ChIP results were confirmed by at least 2 separate experiments.

### Statistical Analysis

Statistical comparisons between multiple groups were analyzed by one-way analysis of variance (ANOVA) followed by a Student-Newman-Keuls test. Where normality and/or equal variance among groups was not met, data were analyzed by a Kruskal-Wallis ANOVA followed by a Student-Newman-Keuls test to detect significant differences. P < 0.05 was considered significant. All statistical analyses were done with the aid of the SigmaStat 3.5 software package (Systat Software, Inc., Point Richmond, CA).

## List of abbreviations

RT-PCR: reverse transcriptase-polymerase chain reaction; EMSA: electrophorectic mobility shift assay; DMEM: Dulbecco's modified Eagle's medium; IPTG: isopropyl-1-thio-D-galactopyranoside; PVDF: polyvinylidene difluoride; LacZ: β-galactosidase; DDS: Denys-Drash syndrome; ChIP: chromatin immunoprecipitation.; BSA: bovine serum albumin.

## Authors' contributions

YM carried out the SRY promoter studies as well as the GATA4/WT1 interaction assays. HT carried out the AMH promoter studies, the transfection experiment in Fig. 4C and the ChIP assay in Fig. 5D. FH performed the DNA-binding experiments. YM and HT analyzed and interpreted the data and generated the initial draft of the manuscript. DWS and RSV conceived of the study, and were responsible for its design and coordination. RSV edited the final version of the manuscript which was read and approved by all authors.

## Supplementary Material

Additional file 1 Species conservation of the consensus GATA and WT1 binding sites in the proximal AMH promoter. Click here for file

Additional file 2 Expression of the renilla luciferase (phRL-TK) control plasmid is not significantly affected by GATA4 and/or WT1 overexpression in HeLa cells. Click here for file
