# Supplementary material for: A GATA4/WT1 cooperation regulates transcription of genes required for mammalian sex determination and differentiation
Source: BMC Mol Biol. 2008 Apr 29;9:44. doi: 10.1186/1471-2199-9-44 (PMC2387164; doi:10.1186/1471-2199-9-44)
Supplement: Additional file 1 — Species conservation of the consensus GATA and WT1 binding sites in the proximal AMH promoter. [file 1471-2199-9-44-S1.pdf]

Additional file 1. Species conservation of the consensus GATA and WT1 binding sites in the proximal *AMH* promoter

Proximal *AMH* Promoter

```
                                                                                          GATA
Mouse  CCTTTGAGACAGTCGCCTCCCACCTGCTGGGCATGAAAAGTG...CCAGGC.ACTGTCCCCCAAGGTCACC....TTTGGTGTTGATAGG
Rat    CTTTTGAGACAGCCGCCTCCCACCTGATGGGCATGCAAAGCGCAGCCAGGC.ACTGT.CCCCAAGGTCACCTCA....GGGGTTGATAGA
Human  TCTTTGAGAA.GGCCACTCTGCCTGGAGTGGGGG......CGC..C.GGGC.ACTGTCCCCCAAGGTCGCGGC.....AGAGGAGATAGG
Bovine .CTTTGAGGGA..CCATAGGCCT..CCCAGGCT.......CACAGC.AGGC.ACCAGCCTTCAAGGTCATGTCCC..AGGAGGAGATAGG
Pig    .TTTTGAGAA..GCCTGCTGGCTTGCCCAGGCT.......CACAGC.AGGCAACCGGCCTCCAAGGTCACATCCC..AGAAGGAGATAGG
               |                   |                       |                       |
             -140                -120                    -100                     -80

                                                                 WT1                        M
Mouse  GGCGTCCCTCCCAAGCAAGCAA......TCTGGCTCAGCCATACATATAAGC.AGGGCCACCCGGACCT.TGCTGTA..CCACCATG
Rat    GGTGTCCCTCCCAAGCAC..........TCTGGCTC..GAATGCATATAAGC.AGGACCACCCAGACCC.TGCCA..TGCCACCATG
Human  GGTCTGTCCTGCA..CAAACACCCCACCTTCCACT...CGGCTCACTTAAGGCAGGC.AGCCCAG.CCCCTGGCAGCACCCACGATG
Bovine GACCGCCC.TGCA..CCACAAACA......GCTC.TGCTCCCTCTTATAAAGTAGGGCAGCCCAG.CCCCTGGAAGCTCCCAGGATG
Pig    GGCTGGCCTCCCA..CACCCACA.....TTCC...TGCTCCCCCATATAAGCCAGGGCAGCCCAG.CCCCTCAAAG.TGCCAGGATG
             |                         |                    |                    |
            -60                       -40                  -20                  +1
```
