# Supplementary material for: A GATA4/WT1 cooperation regulates transcription of genes required for mammalian sex determination and differentiation
Source: BMC Mol Biol. 2008 Apr 29;9:44. doi: 10.1186/1471-2199-9-44 (PMC2387164; doi:10.1186/1471-2199-9-44)

Additional file 2. Expression of the renilla luciferase (phRL-TK) control plasmid is not significantly affected by GATA4 and/or WT1 overexpression in HeLa cells

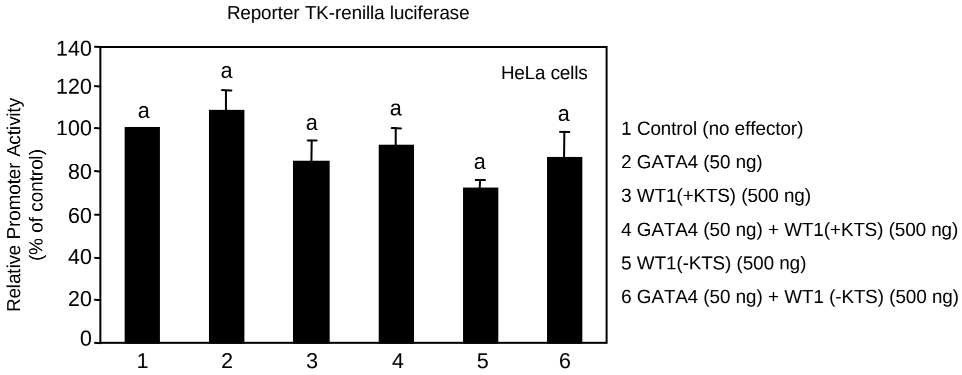

Supplement: Additional file 2 — Expression of the renilla luciferase (phRL-TK) control plasmid is not significantly affected by GATA4 and/or WT1 overexpression in HeLa cells. [file 1471-2199-9-44-S2.pdf]
